# Prosthetic Rehabilitation of a Pediatric Patient with an Ocular Defect

**DOI:** 10.5005/jp-journals-10005-1190

**Published:** 2013-04-26

**Authors:** Triveni Mohan Nalawade, Rachappa M Mallikarjuna, Bina M Anand, Mayur Anand KK Shashibhusan, VV Subba Reddy

**Affiliations:** Reader, Department of Pediatric and Preventive Dentistry, Manubhai Patel Dental College and Hospital, Vishva Jyoti Ashram Near Vishwamitri Bridge, Vadodara, Gujarat, India; Senior Lecturer, Department of Pediatric and Preventive Dentistry KM Shah Dental College and Hospital, Vadodara, Gujarat, India; Senior Lecturer, Department of Prosthodontics, Uttaranchal Dental and Medical Research Institute, Dehradun, Uttarakhand, India; Professor, Department of Prosthodontics, Uttaranchal Dental and Medical Research Institute, Dehradun, Uttarakhand, India; Professor, Department of Pediatric and Preventive Dentistry, College of Dental Sciences, Davangere, Karnataka, India; Department of Pediatric and Preventive Dentistry, College

**Keywords:** Ocular defect, Custom-made ocular prosthesis, Anophthalmos

## Abstract

The eye is a vital organ for vision and an important component of facial expression. Loss of an eye has a crippling effect physically and psychologically. Especially, in case of a child where it affects the parent too and the approach toward these special children needs to be very special indeed. The construction of an ocular prosthesis for a child is the same as for an adult. A growing child will require periodic enlargement of the prosthesis in order to accompany the expansion of the anophthalmic cavity and it is the only way to esthetically rebuild the anophthalmic socket. Although implant eye prosthesis has superior outcome, due to economic factors it may not be advisable in all patients. Therefore, an acrylic custom-made ocular prosthesis replacement as soon as possible is a good alternative to promote physical and psychological healing for the patient and to improve social acceptance. A case of a custom fabricated ocular acrylic prosthesis using the advantages of digital photography is presented here, which had acceptable fit, retention and improved esthetics with a certain degree of motility in coordination with the contralateral normal eye.

**How to cite this article:** Nalawade TM, Mallikarjuna RM, Anand BM, Anand M, Shashibhusan KK, Subba Reddy VV. Prosthetic Rehabilitation of a Pediatric Patient with an Ocular Defect. Int J Clin Pediatr Dent 2013;6(1):62-65.

## INTRODUCTION

Eyes are generally the first facial features to get attention. The loss of ocular globe during childhood due to congenital, traumatic or pathological etiologies affects the growth and development of the orbital area, which may result in hypoplasia, facial asymmetry and adds esthetic and psychological misbalance.^1^ The ocular prosthesis should be provided as soon as possible for the psychological well being of the patient and their parents. Anger, denial and depression set in and the parents of such children suffer great agony. Such special children and their concerned parents, present a challenge for the pediatric dentist that is often unparalled.^2^

This article reports a case of 12-years-old female child treated for an eye prosthesis using digital photography following enucleation of the right eye due to retinoblastoma.

## CASE REPORT

**Fig. 1:**
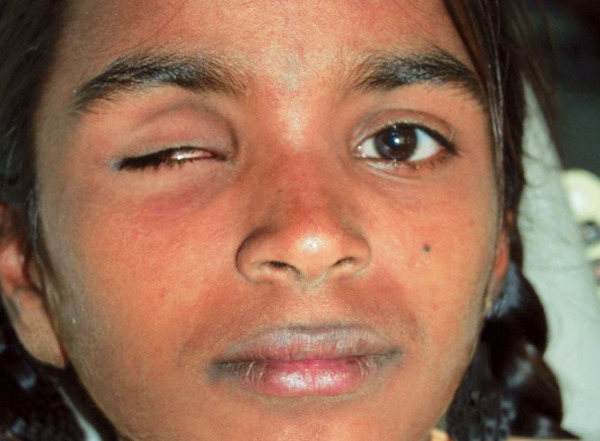
Patient with missing right eye

A 12-year-old female child reported to the Department of Pedodontics and Preventive Dentistry with complaint of missing right eye (Fig. 1). Detailed and careful case history recording revealed that the patient had been diagnosed having retinoblastoma of the right eye and the affected eye had to be enucleated. Patient examination consisting of internal examination of the anophthalmic socket revealed a healthy epithelial lining. Following describes the procedure of eyeball prosthesis. Patient in erect position, seated, to allow the impression of the tissues involved in the defect to record in their natural drape during active posture.

**Fig. 2:**
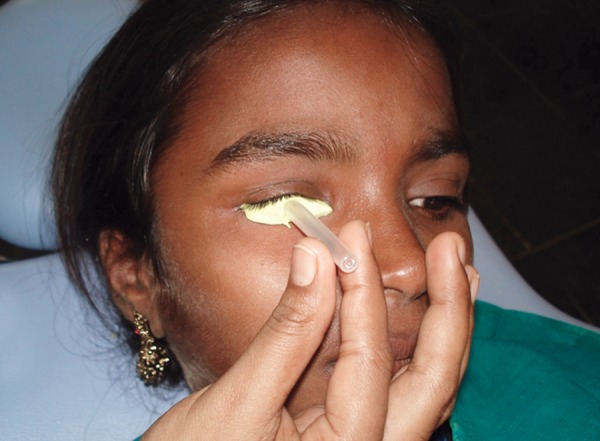
Impression of the socket with light bodied rubber base impression material

**Fig. 3:**
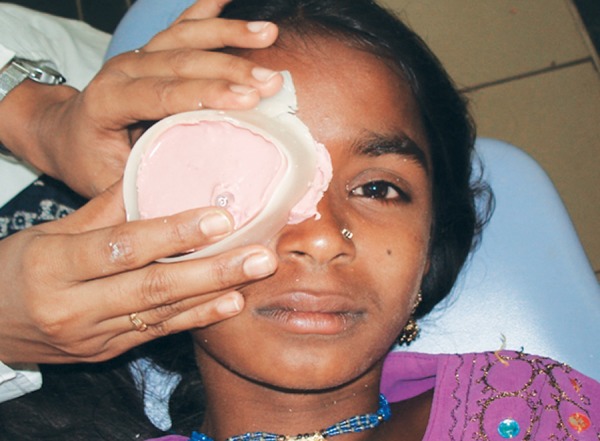
External facial impression made with irreversible hydrocolloid

**Fig. 4:**
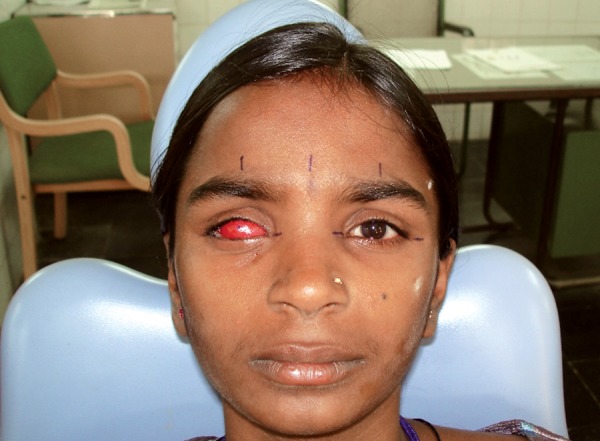
Wax try-in the eye socket

**Fig. 5:**
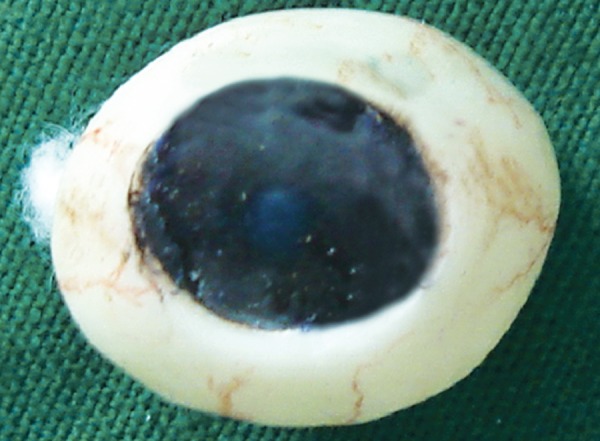
Color characterization and globe completion

**Fig. 6:**
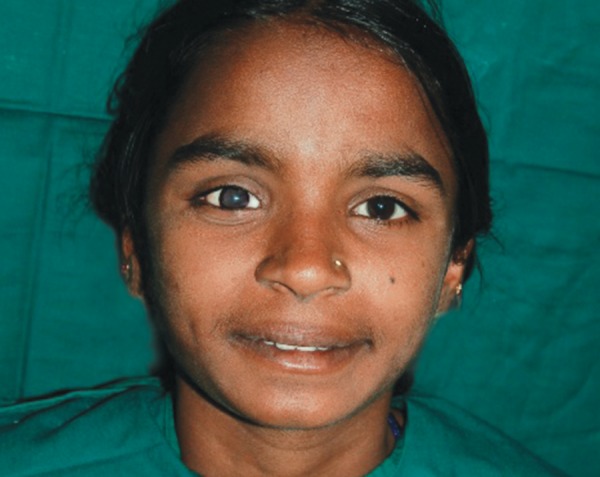
Patient with eye prosthesis

Patient instructed to gaze straight ahead while making the impression of the socket with light bodied rubber base impression material. The impression material was slowly injected into the socket taking care to avoid any air bubbles. The patient was instructed to make various eye movements to get functional impression of the eye. The impression material was reinforced with a syringe needle cover to hold it in place and for ease of removal after it sets (Fig. 2). After boxing the eye region, external facial impression was made with irreversible hydrocolloid (Fig. 3), allowing the material to combine with that of the extruded material, this facilitates the retrieval of the entire impression. In globe formation, a 2-piece dental stone mold was poured to immerse the lower part of the impression. After the stone had set, separating media was applied on the surface. Then the second layer was poured. Grooves were made on all four sides of the cast for proper reorientation of the cast. The impression was separated from the cast of the defect and lubricated the stone cast with a thin coating of vaseline. The lubricated socket of the working cast was filled with molten wax and after solidification; the retrieved wax form was smoothened and polished for try-in on the patient's face (Fig. 4). Vaseline was applied to the tissue surface of the wax pattern to avoid irritation to the tissues, was placed into the clinical defect by introducing it first under the upper lid, and then over the retracted lower lid. Several minutes are required for relaxation of the protective blepharospasm, which may occur when the wax pattern is first placed in the socket. With the wax form in place, and with the patient's eyes closed, both socket areas were palpated simultaneously to compare globe sizes. Modify the wax pattern and its corneal prominence, where necessary, to duplicate the shape of the natural eye and eyelid drape of both eyes was matched. Retract the eyelids and corneal surface was exposed to adjust the wax form to give the best duplication of globe contour. The corrected wax pattern was flasked and processed in tooth-colored acrylic prematched with natural sclera of the unaffected contralateral eye, selected using the tooth-colored acrylic shade guide. Processed resin globe was retrieved from the flasking matrix in a way that preserved the outer stone matrix and later allows reseating of the modified globe back into this matrix for eventual reprocessing of the globe's original contour. In iris characterization, a processed resin globe, with a high polish, placed in the patient's socket for evaluation, and made necessary adjustment to effectively simulate the normal corneal contour as accurately as possible. The patient was instructed to hold an erect position and to gaze straight ahead and observed from the side to determine the iris plane relationship with the normal eye. The distance from the pupil of the normal eye to the midline was used in establishing the horizontal position of the prosthetic pupil center and marked on the globe. The vertical position of the pupil center is determined and marked by the canthus relationships. The diameter of the iris was measured holding a ruler close to the normal steadied eye. The globe form with its marked pupillary center is removed and the iris size that is 1 mm smaller than the diameter of the measured iris is circumscribed with a compass from the established point. Return the globe to the clinical defect and the outlined iris evaluated in relation to that of the eyelids. Accurate iris positioning is critical in the establishment of a natural appearance. The ocular globe modified by cutting away resin within the circumscribed area providing a chamber to house a photographic digital image. In color characterization and globe completion, the iris photographic image of approximately 1 mm smaller than the diameter of the measured patient's iris was cut (Fig. 5) as this will be compensated for, by the magnification caused by the overlay of clear acrylic resin in the completed prosthesis. If necessary, further customization and color modifications are performed using professional quality color pencils. The paper iris was covered with three light coats of water resistant spray and attached to the excavated recess of the globe. The remaining outer corneal surface was characterized by removing a thin layer of acrylic resin and using professional quality color pencils, scleral blood vessels were drawn along the outer periphery. Soft color tones of yellow and brown were added onto the medial canthal area to simulate the normal eye (Fig. 5). Evaluation was done in the patient and the characterized globe form was returned to its original position within the initial flasking matrix. The space created over the disk and between the reduced outer corneal surface of the globe and the stone matrix of the flasking was packed and processed with clear acrylic resin. The retrieved processed ocular globe was trimmed and polished to a high finish using pumice and was critically evaluated for lid drape, contour, iris color and dimension (Fig. 6). The patient was taught to properly insert and remove the appliance and the importance of careful cleansing and handling of the prosthesis was emphasized.

## DISCUSSION

Retinoblastoma is the major cause of ocular globe enucleation during childhood and eye enucleation has been the method of choice in unilateral cases, as reported in the present case. There is fear and anxiety associated with the recurrence of these pathologies; hence, it is very important to know about the details of loss of eye in case history and to be alert during internal socket examination.

The construction of an ocular prosthesis for a child is the same as for an adult. A growing child will require periodic enlargement of the prosthesis in order to accompany the expansion of the anophthalmic cavity and it is the only way to esthetically rebuild the anophthalmic socket.^1,3^ The use of a stock prosthesis needs a large and expensive inventory of ready-made prostheses if an adequate iris selection is desired. The required clinical and laboratory time is not significantly less than the time required to make a custom ocular prosthesis and the result is rarely equal. Fabrication of a custom ocular prosthesis allows infinite variations during construction. Voids that collect mucus and debris, which can irritate mucosa and act as a potential source of infection are minimized. The optimum cosmetic and functional results of a custom ocular prosthesis enhance the patient's rehabilitation to a normal lifestyle.^3^ Beumer et al stated of that a prefabricated resin eye should not be used in eviscerated sockets because intimate contact between the ocular prosthesis and the tissue bed is needed to distribute pressure equally. However, when the prosthesis is customized to the patient using proper impression technique, distribution of pressure should be equal. Intimate adaptation of the custom ocular prosthesis to the tissue surface of the defect uses its full potential to increase the movement of the prosthesis, enhances its natural appearance,^4^ and stimulates the eyelid muscles to move, thus, exercising them and preventing disuse atrophy.^5^ The main disadvantage is the inability to match iris colors and limited variations in iris size. Unfortunately, a pediatric patient is one of few patients that cannot be accommodated by this technique.^4^ Using digital imaging presents several advantages like providing acceptable esthetic results, because it closely replicates the patient's iris with minimal color adjustments and modifications, simplicity, decreases treatment time and requires minimal artistic skills, which are necessary in the iris painting technique.^6^ Only further research is necessary to evaluate the long-term color stability and aging of ocular prostheses.^7^

## CONCLUSION

An ocular prosthesis minimizes the possible discrepancy between the compromised and healthy sides, thus contributing to balance and harmony of the facial development. Hence, the installation of an ocular prosthesis still during childhood adds an inestimable psychological and social contribution to the physical benefit in the patient's global rehabilitation. The extra effort and time put into fabrication of custom-made ocular prostheses has been a boon to patients who cannot afford other alternatives, including implants, and ensures a better drape of lid tissues, and provides a superior natural appearance to both patient and the observer.^8^
